# Magnetic Resonance Microscopy at Cellular Resolution and Localised Spectroscopy of *Medicago truncatula* at 22.3 Tesla

**DOI:** 10.1038/s41598-020-57861-7

**Published:** 2020-01-22

**Authors:** Remco van Schadewijk, Julia R. Krug, Defeng Shen, Karthick B. S. Sankar Gupta, Frank J. Vergeldt, Ton Bisseling, Andrew G. Webb, Henk Van As, Aldrik H. Velders, Huub J. M. de Groot, A. Alia

**Affiliations:** 1 Solid-state NMR, Leiden Institute of Chemistry, Faculty of Science, Leiden University, Einsteinweg 55, Leiden, 2333 CC The Netherlands; 2 Laboratory of Biophysics, Wageningen University & Research, Stippeneng 4, Wageningen, 6708 WE The Netherlands; 3 Laboratory of BioNanoTechnology, Wageningen University & Research, Bornse Weilanden 9, Wageningen, 6708 WG The Netherlands; 4 Laboratory of Molecular Biology, Wageningen University & Research, Droevendaalsesteeg 1, Wageningen, 6708 PB The Netherlands; 5 C.J. Gorter Center for High Field MRI, Radiology department, Leiden University Medical Centre, Leiden University, Leiden, Albinusdreef 2, 2333 ZA Leiden, The Netherlands; 6 Institute for Medical Physics and Biophysics, Leipzig University, Härtelstraße 16/18, Leipzig, 04107 Germany

**Keywords:** Magnetic resonance imaging, Rhizobial symbiosis

## Abstract

Interactions between plants and the soil’s microbial & fungal flora are crucial for the health of soil ecosystems and food production. Microbe-plant interactions are difficult to investigate *in situ* due to their intertwined relationship involving morphology and metabolism. Here, we describe an approach to overcome this challenge by elucidating morphology and the metabolic profile of *Medicago truncatula* root nodules using Magnetic Resonance (MR) Microscopy, at the highest magnetic field strength (22.3 T) currently available for imaging. A home-built solenoid RF coil with an inner diameter of 1.5 mm was used to study individual root nodules. A 3D imaging sequence with an isotropic resolution of (7 μm)^3^ was able to resolve individual cells, and distinguish between cells infected with rhizobia and uninfected cells. Furthermore, we studied the metabolic profile of cells in different sections of the root nodule using localised MR spectroscopy and showed that several metabolites, including betaine, asparagine/aspartate and choline, have different concentrations across nodule zones. The metabolite spatial distribution was visualised using chemical shift imaging. Finally, we describe the technical challenges and outlook towards future *in vivo* MR microscopy of nodules and the plant root system.

## Introduction

Interactions between plants and microbes are considered to be important for the health of the soil ecosystem as a whole^1^. Understanding the metabolic interactions between plants and microbes, both commensal and parasitic, could help address many of the challenges we face today, related to agriculture and food security. One such interaction is the microbiome-mediated uptake of nitrogen by plants. Availability of biologically-active forms of nitrogen in the soil is an important factor determining crop yield. Current agricultural practice, therefore, relies strongly on nitrogen fertiliser to supplement soil nitrogen to ensure high crop yield^2^. The Haber-Bosch nitrogen fixation process, used to produce the ammonia needed for these fertilisers, currently consumes 1% of the world energy sources; making it the most energy consuming process in the chemical industry^3^. In contrast, alternative processes (*e.g*. symbiotic nitrogen fixation, SNF) achieve the same result of fixing nitrogen without the need for high pressure and temperature required by the Haber-Bosch process.

More precisely, plants have solved the problem of biological nitrogen fixation through commensal processes, involving bacterial infection of plant roots^4^. Mutualistic infections are omnipresent in nature, with a wide range of nitrogen-fixing bacteria – such as those that are collectively named rhizobium – invading not just plants but also the phycosphere of green algae^5^. The mutualistic symbiosis involves almost all parts of the plant cell machinery, including plastids and mitochondria. Of particular interest are the interactions between rhizobial bacteria and leguminous plants, which form dedicated organs - root nodules - to accommodate the bacteria. *Medicago. truncatula* infected with *Sinorhizobium meliloti* (*S. meliloti*) has long since been used as a model plant system to study plant-rhizobia interactions^6^. The root nodules facilitate intracellular hosting and nutrient exchange^7^. The result is the efficient formation of fixed nitrogen (NH_4_^+^), which provides considerable growth advantages to leguminous plants^8^. Recently, a detailed fate map of root nodule formation at a genetic level has been developed^9^. It has also been proposed that rhizobial metabolic activity may confer resistance to drought and salt stress^10–12^. More detailed understanding of key metabolites in plant-nodule metabolism and importantly, their localisation within root nodule tissues could, therefore, shed light on the mechanism by which Symbiotic Nitrogen Fixation (SNF) confers advantages to host plants.

Tracking metabolite exchange non-invasively within intact nodule systems is difficult with most imaging modalities, since they require a form of sample fixation, for example in the case of Matrix Assisted Laser Desorption/Ionisation Mass Spectrometry (MALDI-MS) imaging^13^. The current knowledge of the metabolic profile in the root nodules is based on analysis with destructive extraction procedures. For example, Gas Chromatography and Liquid Chromatography in combination with Mass Spectrometry (GC/LC-MS) has provided rich metabolic information but required destructive extraction procedures^14^. These *in vitro* methods may not faithfully reflect the native structural and molecular information. Examining and mapping the levels of metabolites directly in the intact root nodule would be important to understand the functional framework of metabolism in the native state. Magnetic resonance techniques may offer advantages in terms of spatially resolved spectroscopic information on a single nodule, which is difficult to access with alternative techniques. Magnetic resonance imaging (MRI) has been previously used to visualize belowground root structures^15–17^.

Advances in magnetic resonance technology have resulted in steady increase in sensitivity, making Magnetic Resonance Microscopy (MRM) a reality where resolution below 100 μM can be achieved^18^. A combination of advances in gradients strengths, magnetic field strengths and RF coil designs, have made it possible to attain ever higher resolutions^19^. Specifically, microcoils have played an important role in increasing sensitivity, by optimally matching the RF coil to a sample of interest^20–22^. The highest possible resolution on phantom has steadily increased over the years, and thus far maximum nominal resolutions of around 3 μm has been attained on phantoms^23–26^. Biological applications have been diverse, including imaging of neuronal tissue, where cellular resolution were obtained on individual neurons^27–31^. Moreover, highly resolved Nuclear Magnetic Resonance spectroscopy has also been demonstrated using the same type of hardware^32^.

In this work, we applied state-of-the-art magnetic resonance microscopy (MRM) in conjunction with localised spectroscopy at ultra-high magnetic field (22.3 T), using a home-built solenoid RF coil in order to image root nodules of *Medicago truncatula*. We obtained not only cellular level structural information but also mapped some of the metabolic information in localised zones and how they vary across different tissues. Lastly, we describe some of the technical challenges that were encountered and an outlook towards future *in vivo* imaging of nodules and plant root systems.

## Results

### Root nodule morphology resolved by MR microscopy in cellular detail

**Figure 1:**
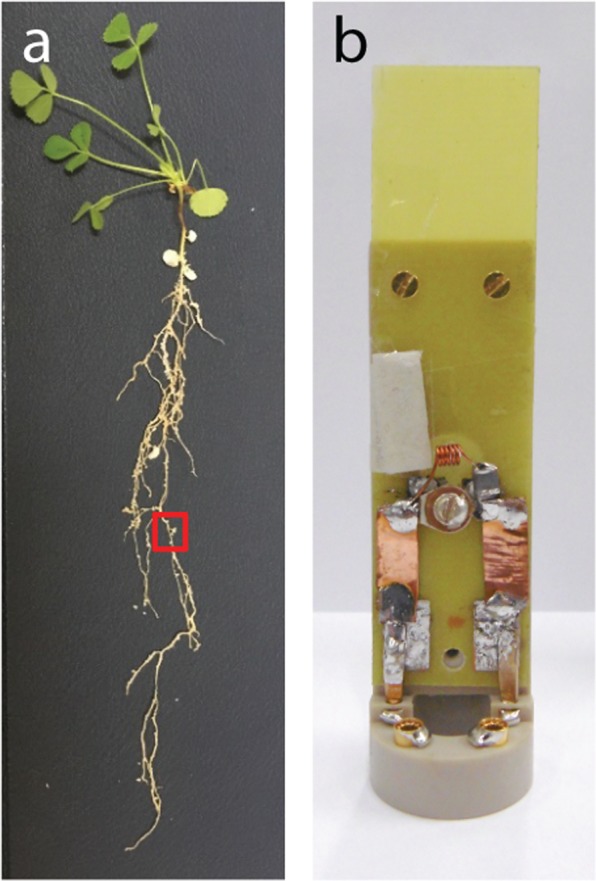
Photograph of M. truncatula root system and custom-designed home-built microcoil. (**a**) Photograph of a representative M. truncatula plant (five weeks old, three weeks post inoculation). Typical root nodule indicated with a red square. Some white perlite beads are still visible along the root system. (**b**) Photograph of a home-built solenoid coil insert mounted on a Bruker insert holder. The solenoid inner diameter is 1.5 mm; further details are described in the materials and methods.

A representative *M. truncatula* specimen with a single root nodule shown in Fig. 1a, was imaged using a custom home-built 1.5 mm diameter solenoid coil as depicted in Fig. 1b.

**Figure 2:**
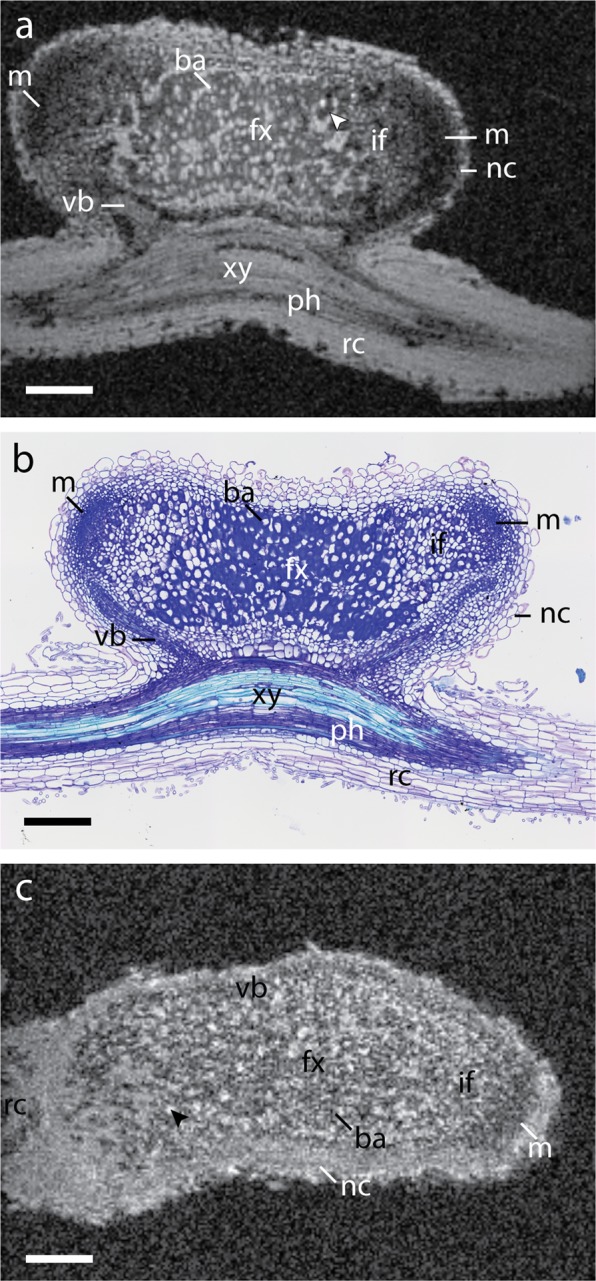
High-resolution MR imaging and optical microscopy of root nodules. (**a**) FLASH image of fixed root nodule at 7 × 7 × 7 µm^3^ resolution with an acquisition time of 34 h24 m. Individual cells containing bacteroids (ba) can be discerned by the dark rings inside the cells, where *S. meliloti* cells accumulate. The nodule exhibits a double meristem (m) on opposed directions with cells becoming smaller towards the meristem until individual cells can no longer be resolved. Air pockets appear as hypo-intense regions marked with an arrowhead. (**b**) Optical transmission microscopy of nodule section (thickness 5 µm) stained with Toluidine blue. (**c**) FLASH image of *in situ* root nodule at 7 × 7 × 7 µm^3^ resolution with an acquisition time of 34 h24 m. Though lower in signal-to-noise, both uninfected cells (black arrow) and infected cells (ba) can be discerned. Abbreviations: rc, root cortex; xy, xylem; ph, phloem; nc, nodule cortex; vb, vascular bundle; fx, fixation zone; if, infection zone; ba, bacteroid containing cells; m, meristem. Scale bars 200 µm.

Individual cells of a root nodule with two lobes and meristems (m) could be distinguished on the MRI scans. A high-resolution 3D Fast Low Angle SHot (FLASH) image of the root nodule, fixed with formaldehyde and submerged in perfluorodecalin (PFD), is shown in Fig. 2a. All image slices through the entire root nodule are shown in the Supplementary Material (Supplementary Video 1). Root nodule morphology in *M. truncatula* nodules are of the indeterminate type, *i.e*., four distinct regions (zones) occur, from the apex of the root nodule to the root attachment point^4^. Nodule tissue - from outward to inward - starting with the nodule cortex (nc), delineated as a high-intensity ring on the surface of the nodule, contrasted with the darker region of the meristem (m). Cells in the meristem, responsible for the growth of the nodule, were significantly smaller than mature bacteroid containing cells. In the next region, the rhizobial infection zone (if), young cells infected by rhizobia bacteria could be seen as a region of alternating high- and low-intensity patches. Not all cells in the rhizobial infection zone were infected, which was apparent due to the presence of variation in image intensities, *i.e*., non-infected plant cells had a higher image intensity as compared to cells infected with the bacterium. Lastly, in the active nitrogen fixation zone (fx), large cells (40 to 50 µm) could be distinguished by their grey rings, caused by the presence of bacteroids surrounding the vacuole^33^. The fixation zone is where rhizobia make nitrogenase, the enzyme required for nitrogen fixation. In the same area, uninfected interstitial cells were visible, which did not exhibit the rings. These interstitial cells perform an important role in the regulation of nitrogen fixation activity^34^. Lastly, connective tissues (vascular bundles) could be seen extending from the root towards the periphery of the nodules, providing a route for nutrient exchange with the rest of the plant.

Figure 2b shows a light microscopy image of a histological slice stained with Toluidine blue, from the same root nodule at approximately the same plane as the MR image (co-registration). Toluidine blue stained the nuclei and lignin of the cell wall^35^. Common features in Fig. 2a,b are seen, including the bacteroid ring as observed on the MR image, occurring in the staining of cells in the nitrogen fixation zone. Especially visible were the vascular bundles extending from the central root, which due to their smaller cells appeared more densely stained in the optical microscopy image. In addition, air pockets could also be imaged as they appeared as hypo-intense regions in the FLASH image (Fig. 2a, arrowhead). Air pockets also introduced differences in magnetic susceptibility within the sample, which influenced image quality. Air pockets of different sizes could be seen within the fixation zone using multiple gradient echo (MGE) imaging with echoes acquired at increasing echo times (Supplementary Fig. 1). Because of the abundance of air pockets in the apical zone, the susceptibility-induced hypointense regions also increased in size in the apical direction.

To evaluate the possibilities of high-resolution MR imaging of root nodule without any fixation or treatment, measurements were performed on freshly excised nodules (Fig. 2c). While the image contrast and signal-to-noise ratio (SNR) was noticeably reduced, individual cells in the nitrogen fixation zone could still be distinguished. The uninfected cells appeared bright while infected cells appeared dark in the active infection zone. Cells in the meristem appear slightly brighter in freshly excised nodule (*in situ*) than in the fixed nodule, possibly due to the native contrast in freshly excised nodule, while in fixed nodule the contrast was possibly affected by formaldehyde fixation^36,37^.

### Localised spectroscopy revealed the spatial distribution of nodule metabolites

**Figure 3:**
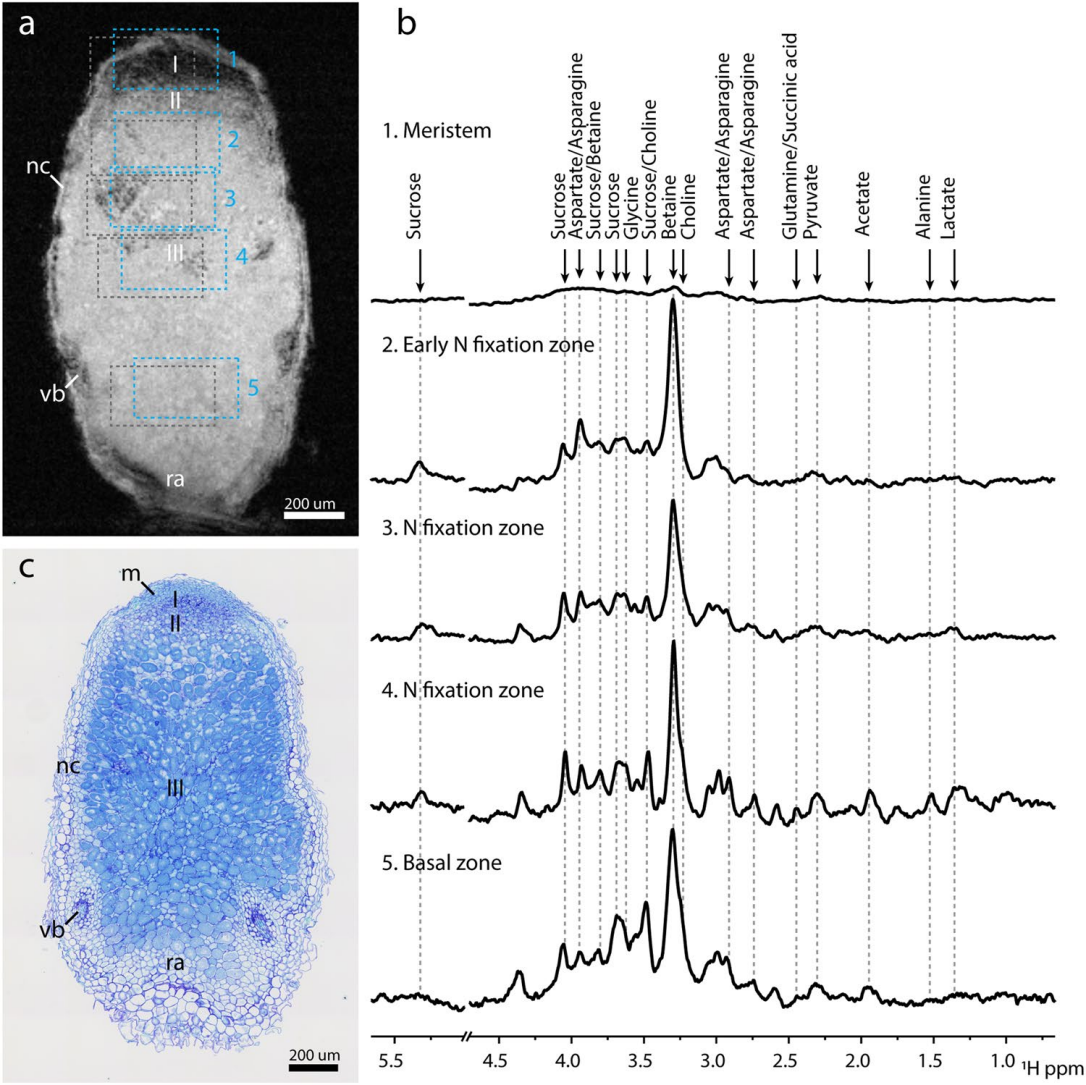
Localised spectroscopy in *in-situ* root nodule reveals sugar differences in pre- and post-granule onset regions. (**a**) FLASH reference with PRESS Region of Interest (ROI), numbered one through five. Roman numerals indicate nodule zones: Meristem (I), Infection zone (II), Nitrogen fixation zone (III). Grey dotted boxes show ROI shift of Betaine due to Chemical Shift Displacement Error (CDSE). Pulse excitation was centred around 4.0 ppm. (**b**) PRESS spectra captured from Regions of Interest (ROI) shown in **a**, for Nodule Meristem (1), (early) Nitrogen fixation zone (2), Nitrogen fixation zone (3 & 4), Basal region (5). PRESS voxel sizes were 200 × 350 × 350 µm^3^. Spectra were aligned to the betaine peak at 3.3 ppm. Water peak region at 4.7 ppm has been omitted for ease of viewing. Intensities of the spectra have not been normalised, reflecting the strength of the signal recorded. This means that for the meristem little information can be discerned. Line broadening 10 Hz. (**c**) Co-registration of Optical Microscopy confirms the presence of bacteroids in the active nitrogen fixation region. Toluidine blue staining 10x magnification. Abbreviations: nc, nodule cortex; vb, vascular bundle; m, meristem; ra, root attachment area. Scale bars 200 µm.

Since the root nodule is an active plant organ whose primary function is to fix nitrogen and to facilitate nutrient exchange, SNF-linked metabolite distribution over the various nodule zones is of interest. According to earlier literature, a nodule may be divided into four zones, based on SNF activity: (I) meristem zone, (II) infection zone, (III) nitrogen fixation zone, and (IV) senescence zone, the latter observed mostly in older nodules. In the infection zone, bacteroids accumulate and mature, while in the fixation zone active SNF takes place^38^. Localised Magnetic Resonance Spectroscopy (MRS) was utilised to distinguish metabolites in a freshly excised and PFD submerged root nodule, based on their frequency differences (chemical shift) and location. Localised MR spectra were recorded from five different regions of interest (ROI), localised in the four different zones in an intact freshly excised root nodule, *in situ* (Fig. 3a). The presence of bacteroids was verified with optical microscopy after the MRI measurements (Fig. 3c). The first ROI was placed on the meristem (ROI 1) in Fig. 3a, where little MR signal was recorded, possibly due to the small volumes of individual cells which may cause T_2_-shortening, reducing the SNR. The second voxel (ROI 2) was placed in the infection/early fixation zone. The third (ROI 3) and fourth voxels (ROI 4) were placed in the largest zone: the active nitrogen fixation zone. The last voxel (ROI 5) was placed in the basal region of the root nodule.

The spectra obtained from ROIs 2–5 showed the presence of several metabolites including various amino acids, sugars, choline and betaine. Betaine was present in a high amount in all four ROIs (Fig. 3b). The signal from sucrose and asparagine/aspartate was also clearly detected. Assignment of various metabolites was confirmed with solution-state NMR performed on an extract obtained from the root nodules, using two-dimensional ^1^H-^1^H correlation spectroscopy (2D-COSY) (see Supplementary Fig. 2). It should be noted that, due to the chemical shift displacement error, which increases with magnetic field strength, the regions of interest chosen for the localised spectra were shifted slightly for each metabolite, as the spatial selection relied on the frequency of the metabolite relative to the chosen centre frequency of 4.0 ppm^39^. This shift meant that the indicated regions of interest in Fig. 3a were correct for signals at δ = 4 ppm, while for example the strongest signal (at δ = 3.3 ppm) originated from an ROI which was shifted by 35 µm along x, 97 µm along y and 97 µm along z (see the dotted region of interest in Fig. 3a).

The strongest peak observed in all spectra was attributed to a resonance of glycine betaine (δ = 3.3 ppm) (Fig. 3b). A complementary spectroscopic imaging method, Chemical shift imaging (CSI) confirmed the presence of betaine throughout the nodule (Supplementary Fig. 3). Betaine serves as an important osmoprotectant produced by *S. meliloti*^40^. Furthermore choline – a precursor to glycine betaine in both plants and bacteria, was visible in ROI 3 as a shoulder on the low frequency side of the betaine peak, and was even more pronounced in ROIs 4 and 5^41^. In uninfected root tissue, choline, but not glycine betaine was detected as seen in solution NMR measurements (Supplementary Fig. 4).

In root nodule, another strong peak from asparagine and aspartate was seen primarily in ROIs 2–5 (Fig. 3b). Notably, glutamine - though present in ROI 4 - appeared much lower in concentration in all ROIs than asparagine, suggesting a smaller pool or higher turnover rate in the nitrogen assimilation process.

Intermediates from the tricarboxylic acid cycle such as acetate (δ = 1.9 ppm), pyruvate (δ = 2.3 ppm) and succinic acid (δ = 2.4 ppm) were also seen in the nitrogen fixation zone (region 3 & 4). Lactate (δ = 1.3 ppm) was visible only in the active nitrogen fixation zone (region 3 & 4). Alanine (δ = 1.5 ppm) has been suggested by metabolic network modelling to be the primary metabolite for the uptake of assimilated nitrogen by plants^42^. However, our results indicated that the pool of alanine was small compared to asparagine.

Sucrose, the most important resource for growth and root nodule activity, was present in all regions with a concentration gradient towards the basal area, starting with the early fixation zone (Fig. 3b, ROI 2). Several peaks were assigned to sucrose (e.g., δ = 5.3 ppm), with some overlap with choline (δ = 3.5 ppm) and fructose (δ = 3.8 ppm). In the basal region (region 5), the sucrose peak at δ = 5.3 ppm was hard to distinguish, possibly due to baseline distortions. Overall, the metabolic pattern exhibited in regions 3–4 appeared to be maintained in the basal region (region 5), though attenuated and with a larger linewidth, possibly reflecting a more disorganised structure of the tissue. At the same time active SNF still appeared to take place, indicating this region is not (yet) the senescent zone IV in which the activity would be reduced.

### Optical microscopic detection of starch correlates with MR based sucrose profile

Starch is a carbohydrate storage polymer that is unique to plants. Indeterminate root nodules are known to form amyloplasts in certain cells, detectable by optical microscopy, especially in the youngest cells of the nitrogen-fixation zone of an actively growing nodule^43^. Accumulation of amyloplasts has also been found in cells located behind the division zone in shoot meristems^44^. Zeeman *et al*. speculated that this might be caused by ‘a temporary imbalance between carbon import and utilisation as cells move from division to expansion and differentiation’^45^. If correct, this marker for high metabolic activity should be reflected in changes to the metabolic profile of bacteroid containing cells. Therefore, we aimed to detect pre- and post-starch granule formation zones in an intact root nodule, and correlate them with sugar metabolites as measured by localised spectroscopy.

**Figure 4:**
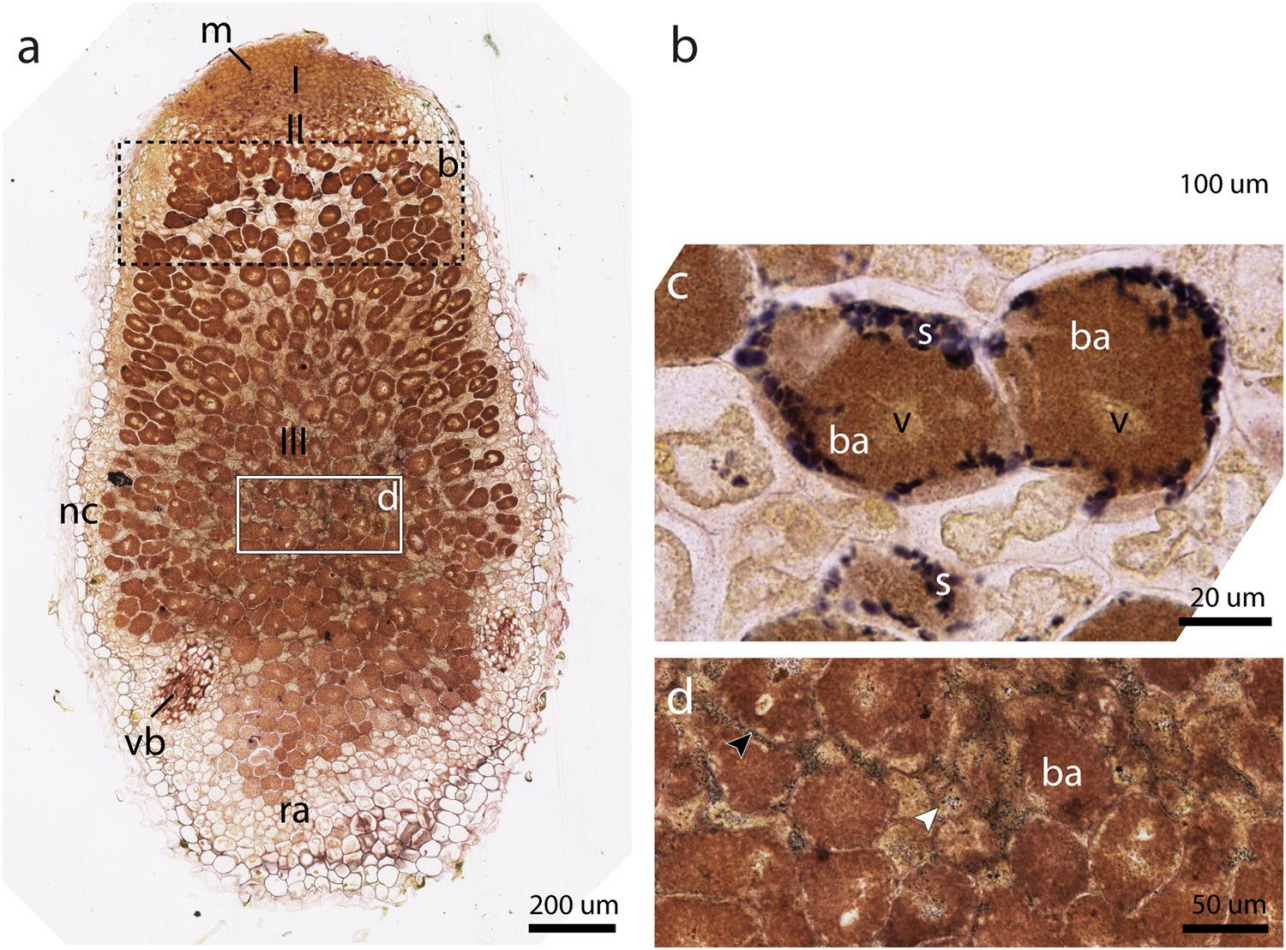
Optical microscopy of root nodules reveals multiple starch distribution patterns. Coupes were stained with both Lugol’s solution and Toluidine blue. (**a**) A section immediately adjacent to that of Fig. 3c reveals starch distribution throughout the nodule. 10x magnification, scale bar 200 µm. A black dotted rectangle indicates the zone shown in (**b**) on an alternate section. A white rectangle indicates an enlargement seen in (**d**). 10x magnification, zoomed; scale bar 50 µm. (**b**) At the first layer of cells in zone III, starch is present as a band (s). A white rectangle indicates the area shown in (**c**). Alternate section, 20x Magnification, scale bar 100 µm. (**c**) Starch granules (s) are located near the periphery of infected cells. The vacuole (v) reappears in the fixation zone after disorganisation in the infection zone. (**b**). 40x Magnification, scale bar 20 µm. (**d**) A fine distribution of starch granules can be seen in Zone III contained in nitrogen fixating cells (black arrowhead) and uninfected cells (white arrowhead). Abbreviations: ra, root attachment site; nc, nodule cortex; vb, vascular bundles; m, meristem; s, starch granules; v, vacuole; ba, bacteroids.

Lugol’s solution was used to stain amyloplasts (Fig. 4a) on an optical section immediately adjacent to that of Fig. 3c, to relate the distribution of sucrose and other metabolites to starch. Starch distribution was seen as dark spots, concentrated in a band located in the first layer of the nitrogen fixation zone as well as in smaller amyloplasts throughout the fixation zone (Fig. 4b,d). In the nitrogen fixation zone, amyloplasts were small and dispersed over infected cells (black arrowhead) as well as uninfected interstitial cells (white arrowhead)(Fig. 4d). In contrast, the cells present in the first layer on the border of the infection zone (II) and fixation zone (III) exhibited a different distribution of starch (Fig. 4b). At higher magnification, large amyloplasts were visible at the periphery of the infected cells, which were almost completely filled with bacteroids (Fig. 4c). Thus, sucrose and starch appear to co-localise with SNF activity. Taken together, starch distribution patterns could provide a possible biomarker for changes in cellular metabolism during nodule development.

## Discussion

This paper showed the feasibility of attaining cellular resolution (7 × 7 × 7) µm^3^ in *M. truncatula* root nodules over a relatively large volume (1.8 × 1.4 × 1.4 mm^3^), opening up new applications for MR microscopy in plant studies (Fig. 2). Individual cells, especially young and mature bacteroids containing cells, were resolved with high contrast in fixed root nodule, exhibiting a unique ‘grey ring’ pattern where *S. meliloti* cells accumulated near the cell walls around a central vacuole. Smaller cells, such as those present in the meristem, could not be resolved individually. A low signal to noise in meristem may be attributed to the shortening of T_1_ and T_2_ relaxation times as a result of formaldehyde-based fixation. Formaldehyde is known to alter relaxation properties of tissues by cross-linking proteins, which may lead to a gel-like cytoplasm. This alters the rates of chemical exchange between water protons and proteins, thus leading to a reduced T_1_ and T_2_^36,37,46^. However, vacuoles are less affected by this, potentially contributing to the strong contrast between bacteroids and vacuoles. Looking towards *in situ* imaging in Fig. 2c, although nodules suffered from lower image contrast and SNR, the meristem appears brighter as compared to the fixed nodule (Fig. 2a). These results also support the notion that lower signal in meristem in fixed nodule could be due to shortening of relaxation parameters. A possible reason for the overall lower image quality in freshly excised nodule may be due to the abundance of air spaces, since no vacuum treatment was applied for *in situ* imaging. More quantitative measurements are required to determine the causes of reduced SNR and contrast.

Although the hypo-intense regions caused by small and large air pockets can be detrimental to overall image quality (Supplementary Fig. 1), they also contain useful information. Air pockets in root nodules of *M. truncatula* are of particular interest, because of the delicate balance of oxygen levels required for the proper functioning of the nitrogen fixation. The pockets may thus have a regulatory role, as nitrogenase is paradoxically both dependent on oxygen for carrying out its function but can also be poisoned by oxygen under high concentrations^47^. Previous MR investigations into root nodules of soybean (*Glycine max*) have focused on investigating the oxygen diffusion barrier, believed to regulate nodule functioning^48,49^. Furthermore, *Glycine max* root nodules are known to be sensitive to changes in gas perfusion^50^. Thus, MRM could be a useful tool to study oxygen regulation mechanisms, due to its ability to detect air spaces through enhanced signal loss.

PRESS localised spectroscopy results show that betaine was distributed throughout the nodule (Fig. 3b). The relatively high concentration of betaine could be due to maintaining a high degree of osmoregulation, an indicator of stress, or both^41^. High asparagine levels seen especially in the nitrogen fixation zone indicate significant SNF activity. The presence of asparagine, as well as betaine, was also confirmed by high-resolution 2D correlation spectroscopy on extracts prepared from nodules (Supplementary Fig. 2). Asparagine, together with glutamine, is known to be a major export product of assimilated nitrogen^51^. Our results show that asparagine accumulates mostly in active SNF areas (regions 3–5). As such, asparagine was absent in normal root tissue (Supplementary Fig. 4).

Metabolite levels of betaine and asparagine might therefore potentially provide useful markers for stress and SNF activity, respectively. The sucrose concentration gradient, decreasing from the basal to the apical end of the nodule, may reflect either consumption rates or limitations in transport through the nodule. In conjunction, starch granules appeared to be localised to two specific regions, a band in the transition from infection to fixation zone, and the fixation zone (III) itself (Fig. 4). The band seemed to provide support for Zeeman’s prediction of amyloplast formation due to a ‘temporary metabolic imbalance’, though it is located at some distance from the meristem (Fig. 4b)^45^. As for the fixation zone, it has been observed in the literature that starch amyloplast accumulation in uninfected cells is related to the expression of SNF-related (*nif*) genes (Fig. 4d)^52,53^. Considering that sucrose was present throughout the nodule, albeit in a diminishing concentration gradient, it is likely that cell-specific metabolism and environmental conditions determine whether and in what quantity starch amyloplasts are formed^38,54^. For instance, sucrose must be metabolised by the host cell in order to provide carboxylic acids that are preferentially taken up by the bacteroids^55,56^. The observed distribution in size and location of amyloplasts could be affected in this way as well.

As an explorative study of ultra-high field MRM for plant imaging, several recommendations might be of interest to the reader. Sample handling and preparation are essential for high-resolution imaging. More generally, special attention needs to be given to managing sample susceptibility at ultra-high fields, since the inclusion of even small air bubbles in the vicinity of samples can distort image quality significantly, as well as cause line broadening in localised spectroscopic experiments. Perfluorodecalin (PFD) was useful as a submerging fluid as its magnetic susceptibility is close to that of tissues and it gives no observable ^1^H signal. It is also efficient in displacing and dissolving air pockets, which are present in root nodule tissue. The absence of extraneous signal allowed for a manual shimming strategy that was sufficient for imaging and provided a good starting point for shimming voxels of interest for localised spectroscopy. Still, maximum shim currents on a particular system may prove to be a limiting factor for micro imaging applications. Another source of sample susceptibility could be the presence of iron in the form of legheamogloblin which regulates oxygen levels. In bacteroids of mature nodules, up to 25% of the total soluble protein consists of legheamoglobin^7^. The high concentrations of this iron-heme containing protein might have implications for the values of longitudinal relaxation (T_1_) and transverse relaxation (T_2_) and their interpretation, as these properties may depend strongly on the physiological state of the root nodule when measured. Thus, with further characterisation, susceptibility-weighted imaging may provide a useful method for evaluating the physiological state of root nodules. Although MRS was done on freshly excised root nodules, the use of PFD as a submerging fluid may have some influence on the vitality of the nodule, since PFD displaces air pockets within the nodule thus, altering oxygen diffusion to the cells.

Rather than a higher resolution per se, the future applications for MRM at ultrahigh field for – *in vivo –* imaging are also enticing, yet challenging. To achieve *in vivo* imaging, novel coil-insert designs with the necessary life support systems would be required. The solenoid coil type used here could be modified to allow for a relatively simple setup that would support whole plants. Vertical-bore MRI systems allow top access to supply light and fresh medium. Additionally, controlled nutrient supply would be desirable, e.g. a continuous medium perfusion setup^57^. Overall, an imaging setup optimised for longitudinal studies would allow testing of stress conditions for host-symbionts, non-destructively.

In conclusion, our results demonstrate that MRM at ultra-high fields in conjunction with microcoils provides a promising technique to determine physiology and metabolic profiling non-invasively in plant root nodules. Further research and development on coil design are required to exploit this application to its full potential.

## Methods

### Growing conditions medicago truncatula

*Medicago truncatula* accession R108 was grown in perlite saturated with Färhaeus medium without nitrate in a growth chamber at a temperature of 21 °C and 16/8-h light/dark cycle^58^. Plants were inoculated with *Sinorhizobium meliloti* Rm41 (OD_600_ = 0.1, 2 mL per plant)^59^. Root nodules were collected for analysis 21 days after inoculation. Perlite was used as a soil replacement to facilitate the extraction of intact root systems.

### Sample preparation

For MRI measurements, a single *M. truncatula* plant was carefully extracted from the perlite substrate. Healthy nodules that exhibited a light-pinkish colour were selected and used for MRI imaging. For *in situ* MRI and MRS measurements, a nodule was excised and was directly used for measurements without fixation. For *ex vivo* MRI and optical microscopy, root nodules were fixed with paraformaldehyde (4% w/v) and glutaraldehyde (5% v/v) in 50 mM phosphate buffer (pH 7.2) at 4 °C overnight, followed by vacuum treatment for 30 minutes. Samples were then stored at a temperature of 4 °C until use.

Using an SZ40 stereomicroscope (Olympus, Tokyo, Japan), individual nodules were selected that could fit within capillaries with an inner diameter of (1050 ± 50) µm (Hilgenberg GmbH, Malsfeld, Germany). To avoid artefacts during MRI acquisition from air bubbles both within and outside the sample, a Perfluorochemical solution (Perfluorodecalin, PFD) was used to submerge the sample. The use of a Perfluorochemical solution not only prevents sample dehydration but also minimizes susceptibility artefacts. PFD was chosen because it has several advantageous properties, making it suitable as an infiltration agent. Importantly, it is a non-toxic compound, capable of dissolving both CO_2_ and O_2_^60^. Furthermore, PFD exhibits a low surface tension (1.9 × 10^−2^ N m^−1^), below the limit for stomatal penetration (2.5–3.5 × 10^−2^ N m^−1^) in *Arabidopsis thaliana* leaves^60,61^, making it useful for reducing air bubbles within the sample without entering the cells. Care was taken to minimise the required amount of PFD as it is known to function as a potent greenhouse gas^62^.

During insertion of the nodule in the centre position of the capillary, both nodule and capillary were kept submerged in PFD. Capillary wax (Hampton Research, California, USA) was then applied to both ends of the open capillary with a MaxWax Pen (Hampton Research, California, USA). Considerable effort was spent on minimising the inclusion of air bubbles within the capillary.

For solution NMR measurements, metabolites were extracted from root and root nodules as described in detail by Kim *et al*. (2010)^63^. Key steps included manual excision of root and root nodule tissue and consequent crushing with mortar and pestle in the presence of liquid nitrogen. Samples were then freeze-dried and lastly extracted with an extraction buffer consisting of 50% (v/v) deuterated methanol and 50% D_2_O phosphate buffer (pH 6)^63^.

### MRI measurements

A custom solenoid microcoil was built in order to achieve the necessary sensitivity required for high-resolution imaging. For the microcoil assembly, a glass-fibre circuit board was used as a base to hold all the necessary components in place. The radiofrequency (RF) coil consisted of coated 28 AWG (Ø 0.4 mm) copper wire, wound into a solenoid with six windings around a glass capillary with an outer diameter of (1500 ± 50) µm. A fixed 1.5 picoFarad (pF) capacitor was placed in series with the RF coil. To complete the resonant circuit, a single 1.5–6 pF variable capacitor was placed parallel to the RF coil and 1.5 pF capacitor, which allowed for fine-tuning the resonant frequency to 950 MHz. The circuit board was then attached to a Micro5 probe (Bruker Biospin, Ettlingen, Germany) compatible circuit holder.

MRI measurements were performed on a Bruker 22.3 T (950 MHz) spectrometer (Bruker Biospin, Ettlingen, Germany), at the uNMR-nl national facility (Utrecht, The Netherlands). The magnet had a vertical bore of 54 mm in diameter and was connected to an Avance III HD console. A Bruker Micro5 probe with exchangeable RF inserts allowed operation of the 1500 µm custom-built solenoid resonator. The Micro5 probe contained a built-in 48 mT/m/A (2.88 T/m at 60 A) triple axial gradient system coupled to GREAT 60 A amplifiers and cooled with a BCU 20 water cooler. Control of spectrometer operations was performed with Paravision 6.0.1 and Topspin 3.1PV running on a CentOS workstation. Sample temperature was maintained at (298 ± 1) K using a BCU II cooler.

High-resolution 3D Fast Low Angle Shot (FLASH) images on *ex vivo* and *in situ* root nodules were acquired with the following parameters: matrix size 256 × 192 × 192; read direction along the largest matrix direction; the field of view was (1.8 × 1.4 × 1.4) mm^3^. Resolution (7 × 7 × 7) µm^3^ isotropic, number of averages 28, acquisition time 34 h 24 m, repetition time 120 ms, flip angle 30° and echo time 2.9 ms. Receiver bandwidth was set to 100 kHz. To allow for co-registration with coupes from optical microscopy, the JIVE tool was utilised to generate oblique slices.

### Localised Spectroscopy

Point Resolved Spectroscopy (PRESS) was used for localised magnetic resonance spectroscopy (MRS) on root nodules^64,65^. PRESS employs 90°-180°-180° orthogonal pulses with concurrent slice selective gradients. The PRESS sequence was preceded by a VAPOR (Variable Pulse Power and Optimized Relaxation Delays) sequence for global water suppression, which consists of seven variable power RF pulses with an optimised relaxation delay^66^. Prior to MRS, a reference FLASH image was acquired with the following parameters: echo time 3.3 ms; repetition time 80 ms; matrix 192 × 128; field of view 1.8 × 1.2 mm and receiver bandwidth 50 kHz; in plane resolution 9 × 9 µm; slice thickness 52 µm; number of averages 256; and acquisition time 43 m. Appropriate volumes of interest (voxel) for PRESS were selected from the reference FLASH image. Correct positioning of the voxel was also verified after all spectroscopic measurements, by high-resolution 2D FLASH. Five individual MRS voxel (200 × 350 × 350 µm^3^) were localised covering various zones in the nodule. The local field homogeneity was optimised over the voxel by MAPSHIM. The field homogeneity was further improved by manual shimming of up to second order shims (Z-Z^2^-Z-X-Y-Z-Z^2^-Z-XY-XZ-YZ-Z). The time needed for shimming varied between 5–10 min. The field homogeneity resulted in a water line width of 10 Hz. For PRESS measurement, repetition time was set to 1000 ms, echo time was 7.2 ms, and 2048 averages were acquired for a total acquisition time of 34 m 8 s. Spectra were manually phased and automatically baseline corrected with a 3^rd^ or 6^th^ order Bernstein-polynomial fit. Chemical shift alignment was adjusted to the largest peak (3.3 ppm).

### Chemical shift imaging

Chemical Shift Imaging (CSI) was used to acquire a multi-voxel spatial map of metabolic distribution. A large volume of interest was selected using a PRESS approach, consisting of three mutually orthogonal slice selective pulses. Then phase encoding occurred in two directions by applying incremental pulse gradients in each direction, resulting in a matrix of spectroscopic spin echoes.

The following basic parameters were used: Repetition time = 760 ms; echo time = 3 ms; Matrix = 24 × 16; FOV = 1.8 × 1.2 mm^2^; slice thickness = 400 µm; resolution = 150 × 150 × 400 µm^3^; number of averages = 1024. Spectra were acquired with 2048 points; dwell time 26.4 µs; spectral width 19.93 ppm (18,939 Hz). Magnetic field homogeneity in the selected volume was optimized by shimming the water resonance. A VAPOR suppression scheme was applied for efficient water signal saturation before CSI acquisition.

Reference FLASH images were acquired with the following parameters: matrix size was 96 × 64; Read direction along the largest matrix direction; field of view was 1.8 × 1.2 mm^2^. Slice thickness was 0.1 mm; resolution (19 × 19 × 100) µm^3^ isotropic. Number of slices 4; number of averages 128; acquisition time 28 m 40 s. Repetition time was 210 ms; flip angle 30° and echo time 3.26 ms. Receiver bandwidth was set to 50 kHz.

For the processing of the CSI data, the integration of selected signal of specific metabolite areas in magnitude mode was overlaid on reference FLASH images using the Bruker CSI Visualisation Tool.

### NMR measurements

NMR measurements were performed on a Bruker 20.0 T (850 MHz) spectrometer with a vertical bore 54 mm in diameter connected to an Avance III HD console. A triple-tuned broadband cryoprobe was used. Topspin 3.2 was used to control the spectrometer and for processing of the acquired data. The sample temperature was maintained at 298 K.

^1^H NMR spectra were collected using a zgpr30 sequence with pre-saturation to suppress water efficiently. Data was acquired into 65k points; dwell time 29.3 µs, spectral width 20 ppm or 17,045 Hz; number of averages, 512; dummy scans 4; pre-scan delay 10 µs. 2D homonuclear ^1^H-^1^H experiments were performed using chemical shift correlated spectroscopic sequence (COSY). The following parameters were used: 3400 points in the direct dimension, 400 points in the indirect dimension in Digital Quadrature Mode. Both dimensions zero-filled to 4096 points. Number of averages, 96; dummy scans, 16; Spectral width 12.0 ppm, 10,000 Hz; Temperature 298 K.

Assignments were cross-checked against metabolite databases and fitting was performed using Chenomx deconvolution software (Chenomx, Edmonton, Canada)^67,68^. 1D reference spectra were acquired with parameters as follows: data was acquired into 65k points; dwell time 41.6 µs, spectral width 20 ppm or 12,019 Hz; number of averages, 64; dummy scans 4; pre-scan delay 10 µs. COSY reference spectra were acquired with the following parameters: 4096 points in the direct dimension, 256 points in the indirect dimension in Digital Quadrature Mode. Indirect dimension zero-filled to 4096 points. Number of averages, 4; dummy scans, 16; Spectral width 12.3 ppm, 7200 Hz; Temperature 298 K.

All experimental data were acquired and processed using Paravision 6.0.1 (Bruker Biospin, Ettlingen, Germany) and Topspin 3.1 running on Linux. The default bi-cubic interpolation was applied to enhance the details of the image prior to exporting images. Figures were prepared in Adobe Photoshop CC 2019 and Adobe Illustrator CC 2019 (Adobe Systems Incorporated, Mountain View, California, USA). Stacked spectra were produced in MestReNova (MestreLab Research S.L., Santiago de Compostela, Spain).

### Light microscopy

After MR imaging, the sample material was dehydrated in an ethanol series and subsequently embedded in Technovit 7100 resin (Heraeus Kulzer, Hanau, Germany) according to the manufacturer’s protocol. Sections (5 µm) were made by using a microtome (Reichert-Jung, Leica Microsystems, Netherlands), stained with 0.05% Toluidine blue O for 1 min and additionally with Lugol’s iodine solution for a few seconds (if applicable). Sections were analysed by using a DM5500B microscope equipped with a DFC425C camera (Leica Microsystems, Wetzlar, Germany).

## Supplementary information

Supplementary information.

Supplementary video 1.
