# Supplementary material for: Magnetic Resonance Microscopy at Cellular Resolution and Localised Spectroscopy of Medicago truncatula at 22.3 Tesla
Source: Sci Rep. 2020 Jan 22;10:971. doi: 10.1038/s41598-020-57861-7 (PMC6976659; doi:10.1038/s41598-020-57861-7)
Supplement: Supplementary file 1 — Supplementary information. [file 41598_2020_57861_MOESM1_ESM.docx]

# Supplementary Information

Magnetic Resonance Microscopy at Cellular Resolution and Localised Spectroscopy of *Medicago truncatula* at 22.3 Tesla

Remco van Schadewijk^1^, Julia R. Krug^2,3^, Defeng Shen^4^, Karthick B.S. Sankar Gupta^1^, Frank J. Vergeldt^2^, Ton Bisseling^4^, Andrew G. Webb^5^, Henk Van As^2^, Aldrik, H. Velders^3^_,_ Huub J.M. de Groot^1^, A Alia^1,6*^

^1^Solid-state NMR, Leiden Institute of Chemistry, Faculty of Science, Leiden University, Leiden, Einsteinweg 55,2333 CC, the Netherlands.

^2^Laboratory of Biophysics, Wageningen University & Research, Wageningen, Stippeneng 4, 6708 WE, the Netherlands.

^3^Laboratory of BioNanoTechnology, Wageningen University & Research, Wageningen, Bornse Weilanden 9, 6708 WG, the Netherlands.

^4^Laboratory of Molecular Biology, Wageningen University & Research, Wageningen, Droevendaalsesteeg 1, 6708 PB, the Netherlands.

^5^C.J. Gorter Center for High Field MRI, Radiology department, Leiden University Medical Centre, Leiden University, Leiden, Albinusdreef 2, 2333 ZA Leiden, The Netherlands.

^6^Institute for Medical Physics and Biophysics, Leipzig University, Leipzig, Härtelstraße 16/18, 04107, Germany.


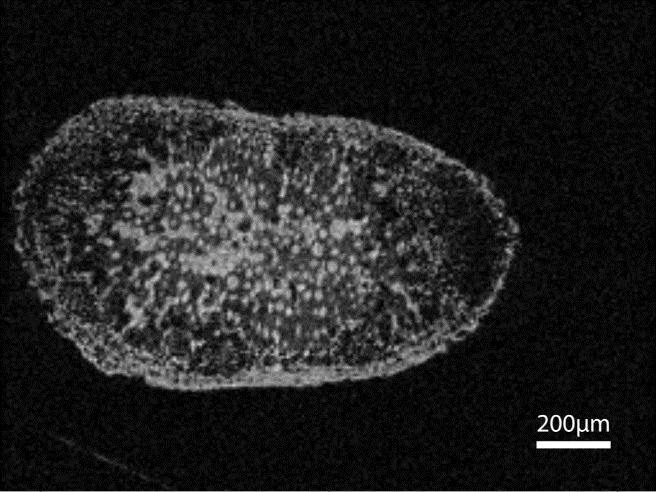


**Supplementary Video 1.** High-resolution 3D FLASH video of fixed and vacuum treated root nodule. Sliced along original matrix orientation. Aspect ratio 4:3, video resolution 1036x768 pixels, 25 frames per second. Duration 6.4 s, 160 frames. Scale bar 200 µm.


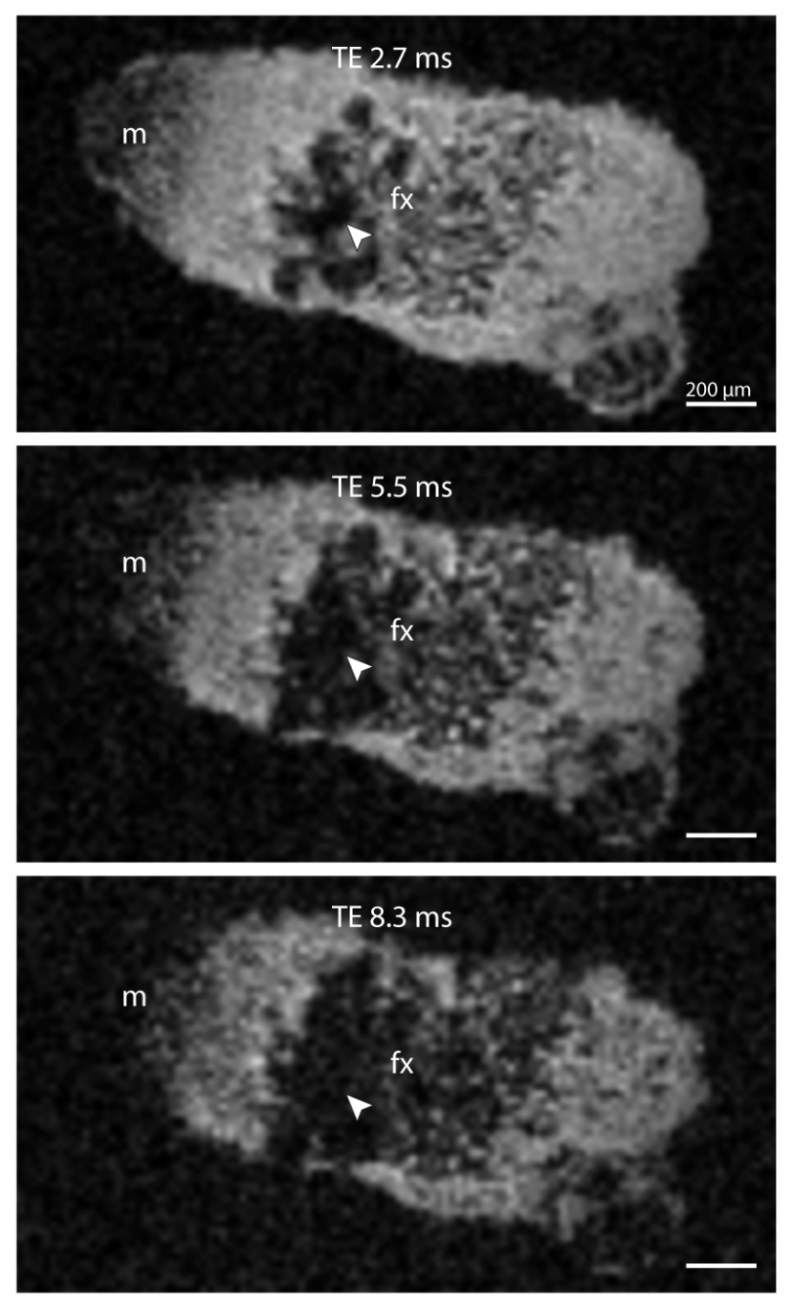


**Supplementary Figure 1.** T_2_-weighted images reveals susceptibility artefacts caused by air pockets. An MGE sequence was used to acquire scans with increasing echo times on a fixed but not vacuum treated nodule. A total of 32 gradient recalled echoes were acquired; initial echo was 2.7 ms, further echoes were spaced 2.8 ms apart resulting in echo times of 2.7, 5.5, 8.3, …, 89.5 ms. Matrix size was 128×64×64; Read direction along the largest matrix direction; field of view was (1.8×1.4) mm^2^. Slice thickness was 1.4 mm; resolution (19×19×19) µm^3^. Number of averages was 24; acquisition time 3 h 16 m. Repetition time was 120 ms; flip angle 5°. A progressive loss of signal as a function of echo time can be seen in the images. Nodule Apical-Basal orientation is left-to-right. Dark areas are indicative of air pockets that dephase signal through increased local magnetic susceptibility differences (arrow). Notably, air pockets are variable in size with smaller pockets seen in the basal direction. The meristem and nitrogen fixation area are particularly affected by the signal loss, though the meristem signal loss is likely due to short T_2_, not susceptibility. Abbreviations: fx, fixation zone; m, meristem. Scale bar 200 µm.


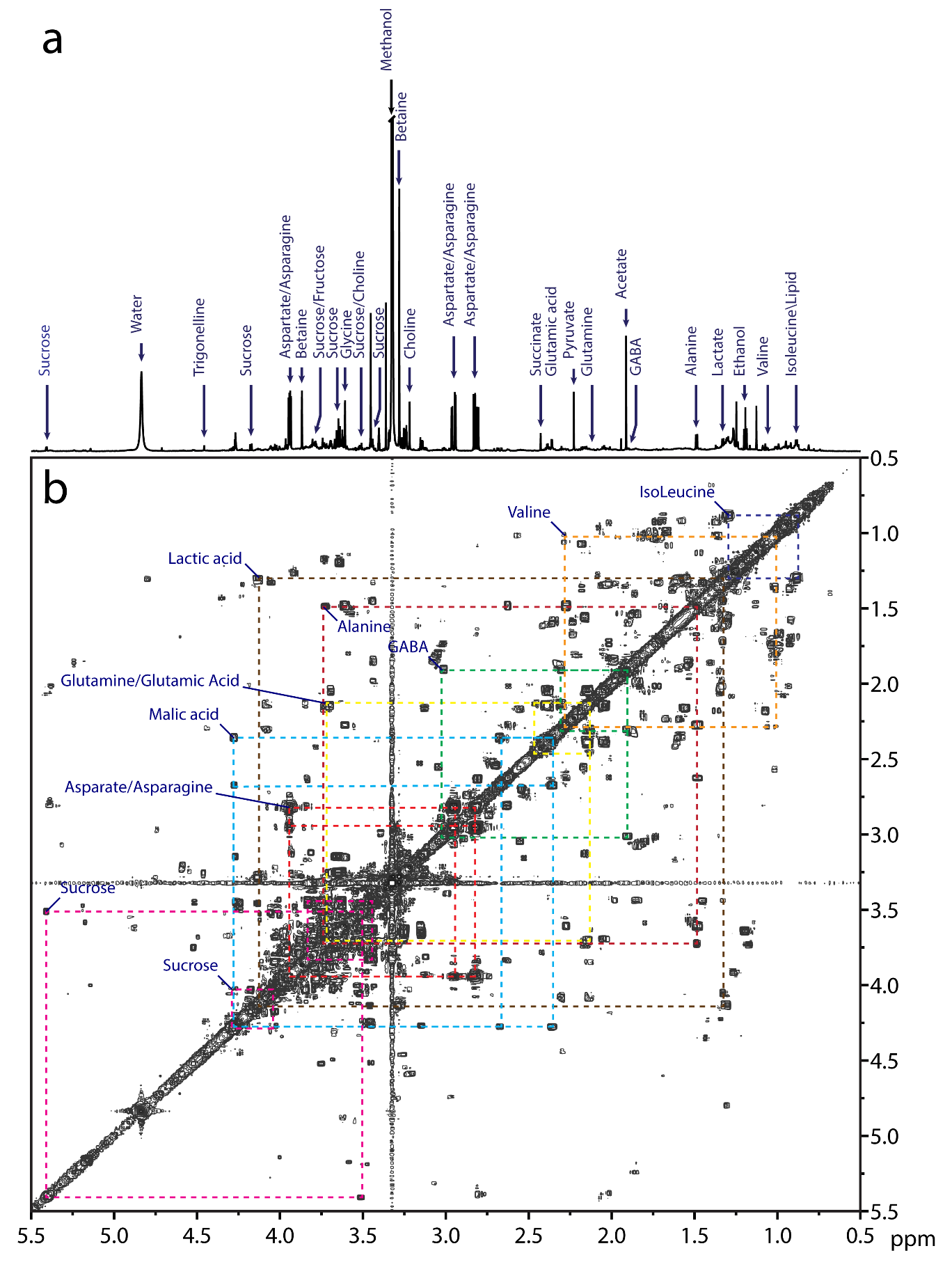


**Supplementary Figure 2.** Homo-nuclear ^1^H correlation spectroscopy (COSY) of root nodule. (**a**) 1D NMR from 0.5 to 5.5 ppm with the most abundant metabolites assigned. Solvent Methanol/D_2_O, water suppressed. GABA = γ-aminobutyric acid. (**b**) COSY was used to verify assignments of in situ PRESS results. COSY assignments for the most relevant metabolites are indicated with coloured boxes. Range 0.5 to 5.5 ppm, water suppressed.


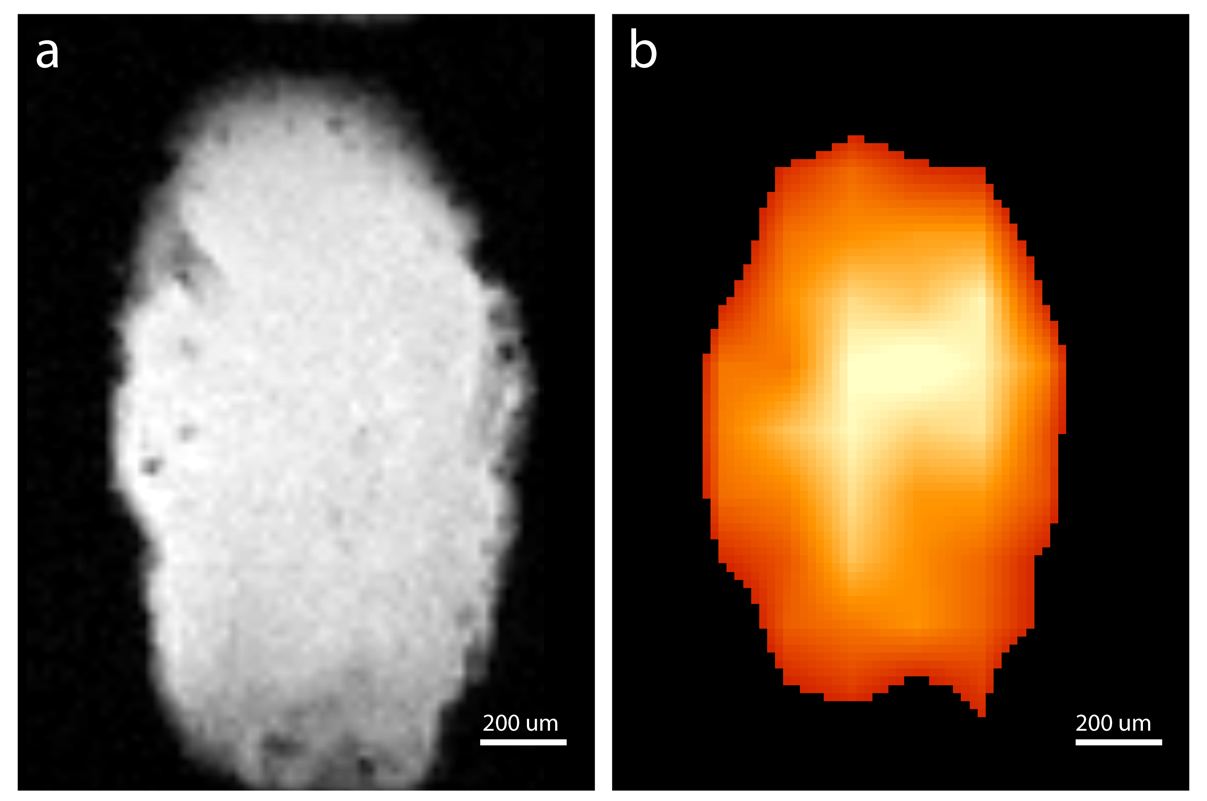


**Supplementary Figure 3.** Chemical Shift imaging showing betaine localisation. Nodule Apical-Basal orientation is top to bottom. (**a**) MSME reference image. Note that the CSI volume of interest is larger than the nodule itself. Thus, the distribution of metabolites may be skewed. (**b**) Heat map generated from betaine peak (integration bandwidth 300 Hz).


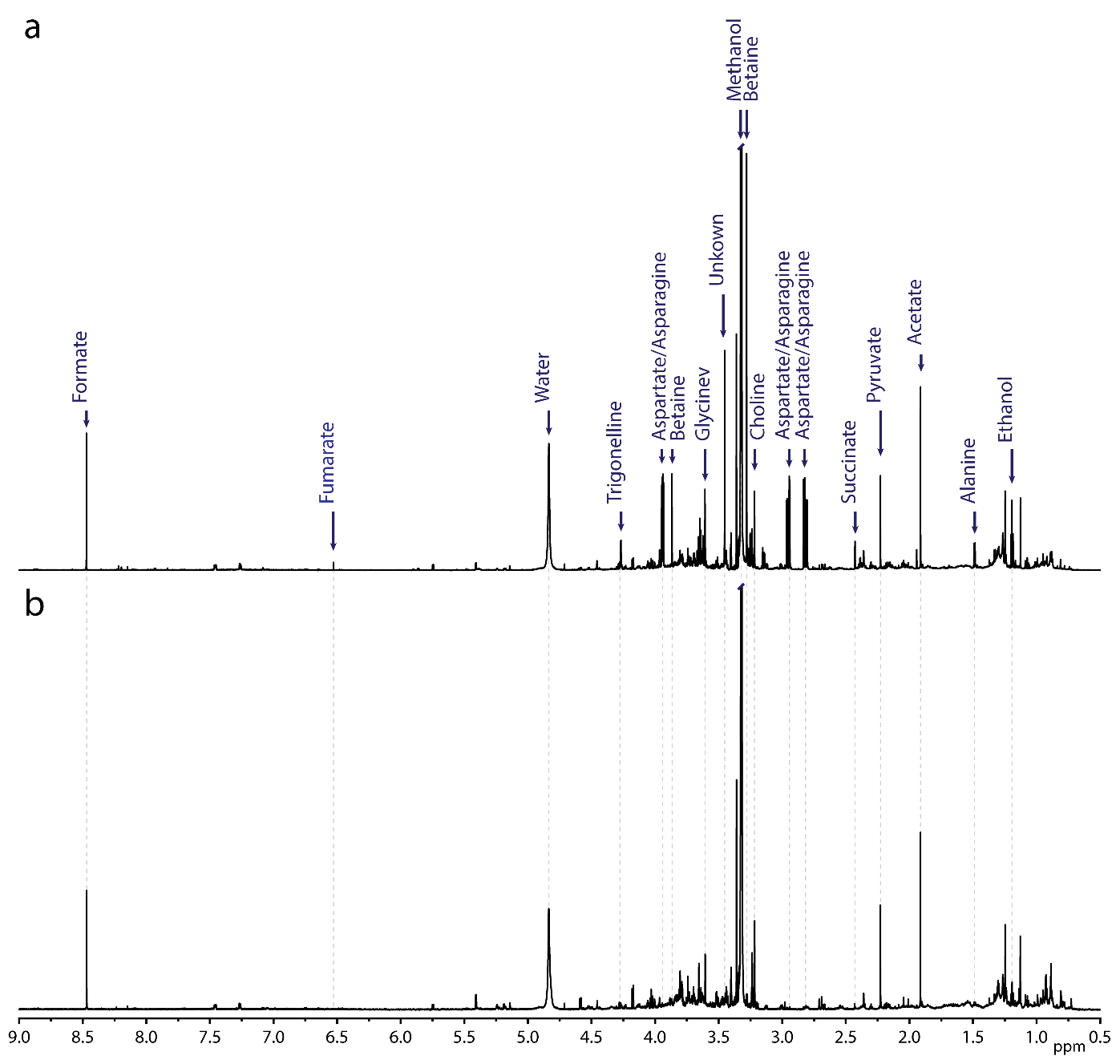


**Supplementary Figure 4.** A representative solution state ­^1^H NMR spectrum of root and nodule tissue, revealing differences in several major metabolites. Spectra acquired at 850 MHz. **(a)** Nodule tissue **(b)** Root tissue.
